# 64385 Patient Reports of New Diagnosis Compared to Electronic Medical Record Documentation following Emergency Department Visit

**DOI:** 10.1017/cts.2021.735

**Published:** 2021-03-30

**Authors:** Kelly Gleason, Susan Peterson, Cheryl Himmelfarb, David Newman-Toker

**Affiliations:** 1 Johns Hopkins University School of Nursing; 2 Johns Hopkins University School of Medicine

## Abstract

ABSTRACT IMPACT: We conducted a study to understand how a patient’s report of a new diagnosis compares with what was documented in the electronic medical record, since it is critical to the diagnostic process that the patient both understands and agrees with a new diagnosis. OBJECTIVES/GOALS: We sought feedback on patient’s understanding of their diagnosis and health status follow Emergency Department discharge. We compared patient report of a new diagnosis to documentation in the electronic medical record. METHODS/STUDY POPULATION: To compare patient reported diagnoses to documented diagnoses, we employed a longitudinal cohort study design at 3 of emergency departments in an academic health system in the Mid-Atlantic. Patients consented to complete questionnaires regarding their understanding of their diagnosis and/or follow-up steps and their health status at 2 weeks, 1 month, and 3 months following emergency department discharge. Inclusion criteria: adult ED patients aged 18 and older seen within the last 7 days with one or more of the following common chief complaints: chest pain, upper back pain, abdominal pain, shortness of breath/cough, dizziness, and headache. We compared patient report of a new diagnosis following discharge to documentation in the electronic medical record. RESULTS/ANTICIPATED RESULTS: Of the sample recruited (n=137), the majority were women (66%, n=91), the average age was 42 (SD 16). A third (n=45) were black and 56% (n=76) were white. The majority of participants (84%, n=115) reported that they either understood the diagnosis they received on ED discharge, or were not given a diagnosis but they understood follow-up steps. At two weeks following discharge, 25% of participants (n=36) had a new diagnosis identified after discharge and 33% (n=45) reported that their health status stayed the same or worsened. There was 85% agreement (kappa 0.49) between patient report of a new diagnosis and a new diagnosis identified in the electronic medical record. Only one of the participants who reported a new diagnosis also reported seeking healthcare outside of the health system. DISCUSSION/SIGNIFICANCE OF FINDINGS: Patient report of a new diagnosis following emergency department discharge had moderate agreement with new diagnoses identified in the electronic medical record, and differences in agreement were not explained by outside healthcare visits.

